# Application-Oriented Marine Isomerases in Biocatalysis

**DOI:** 10.3390/md18110580

**Published:** 2020-11-21

**Authors:** Antonio Trincone

**Affiliations:** Institute of Biomolecular Chemistry, National Research Council, Via Campi Flegrei, 34, 80078 Pozzuoli, Italy; antonio.trincone@icb.cnr.it

**Keywords:** marine enzymes, biocatalysis, marine biotechnology

## Abstract

The class EC 5.xx, a group of enzymes that interconvert optical, geometric, or positional isomers are interesting biocatalysts for the synthesis of pharmaceuticals and pharmaceutical intermediates. This class, named “isomerases,” can transform cheap biomolecules into expensive isomers with suitable stereochemistry useful in synthetic medicinal chemistry, and interesting cases of production of l-ribose, d-psicose, lactulose, and d-phenylalanine are known. However, in two published reports about potential biocatalysts of marine origin, isomerases are hardly mentioned. Therefore, it is of interest to deepen the knowledge of these biocatalysts from the marine environment with this specialized in-depth analysis conducted using a literature search without time limit constraints. In this review, the focus is dedicated mainly to example applications in biocatalysis that are not numerous confirming the general view previously reported. However, from this overall literature analysis, curiosity-driven scientific interest for marine isomerases seems to have been long-standing. However, the major fields in which application examples are framed are placed at the cutting edge of current biotechnological development. Since these enzymes can offer properties of industrial interest, this will act as a promoter for future studies of marine-originating isomerases in applied biocatalysis.

## 1. Introduction

In 2010, one of the first comprehensive review articles about enzymes of marine origin especially suitable for future applications in biocatalysis reported an account of the knowledge in the field. As stated in the conclusion, the enormous pool of marine biodiversity is an excellent natural reservoir for acquiring an inventory of enzymes that is one of the focal points of the potential of blue biotechnology. The importance of the examples reported, picked up from the different classes of enzymes, supported the view that the marine environment is to be considered as an additional source of new enzymes for the biocatalysis. Important examples are among oxidoreductases and carbohydrate-active enzymes. Their novel chemical and stereochemical properties are included in the list of useful habitat-related characteristics possessed by these enzymes, adding value to the often-observed usual resistance of these proteins to high salt concentration and/or organic solvent. Other enzymes, characterized by a potent chemical action on nonactivated carbon atoms are also of extreme interest. Details on the characteristics of other marine representatives belonging to other classes of enzymes (lipid active hydrolases, novel esterases, and other hydrolytic activities) further supported the conclusion [1]. Isomerases are included only with a few cases, in particular, an alanine racemase from the hepatopancreas of the black tiger prawn, *Penaeus mondon* [2] and a fatty acid isomerase from marine alga *Ptilota filicina* [3].

Moreover, in a subsequent review, dated 2016 [4], on the advances in marine enzymes used in food and pharmaceutical applications, although the list of enzymes discussed included oxidoreductases, hydrolases, transferases, ligases, lyases, and explicitly mentioned isomerases, no details on examples of the latter class were reported.

Among the seven major groups of the international classification of enzymes, the class EC 5.xx and subclasses include isomerases, a group of biocatalysts that interconvert optical, geometric, or positional isomers. These enzymes, involved in the central metabolism of most living organisms, also have trivial names, such as epimerase, racemase, *cis*-*trans* isomerases, cycloisomerase, and tautomerase, according to the specific reaction types they catalyze. One of the illustrative examples is triose phosphate isomerase (5.3.1.1) that catalyzes the interconversion of dihydroxyacetone phosphate and d-glyceraldehyde 3-phosphate. In practical examples reported for the applications of isomerase in the field of biocatalysis, some important molecules are prepared. In a recent review for the biocatalyzed synthesis of pharmaceuticals and pharmaceutical intermediates [5], the cases reported concern the transformation of cheap biomolecules into expensive isomers possessing suitable stereochemistry for applications in synthetic medicinal chemistry, as described for production of l-ribose (l-arabinose isomerase from *Paenibacillus polymyxa*), d-psicose (cascade reactions with thermophilic enzymes), lactulose (cellobiose 2-epimerase from *Caldicellulosiruptor saccharolyticus*), and d-phenylalanine (cascade reaction using d-succinylase from *Cupriavidus* and N-succinylamino acid racemase from *Geobacillus stearothermophilus*). In addition, the use of racemases at the industrial level for chiral resolution of racemates is mentioned. In these processes, the racemases are usually part of a cascade reaction and other enzymes are responsible for the chiral resolution [6]. In their conclusions, the authors recognized the widespread distribution of isomerases among species with high biodiversity, indicating that isomerases suitable for many synthetic problems can be identified in particular habitats.

Economically successful biocatalytic processes depend on robust performance, high selectivity, increased stability, increased activity, and broader availability of biocatalysts. Marine-originating enzymes can offer most of these features. Environmental concern is the main factor to drive the growth of the industrial enzymes market globally (projected to reach USD 8.7 billion by 2026). In this, growing attention to green technologies for the conversion of biomass and saccharification of carbohydrate polymers isomerases are included for starch and dairy industries.

The current interest for application-oriented isomerases in scientific literature is demonstrated in the field of rare sugars applied as sweeteners and building blocks as in the study of ribose-5-phosphate isomerase of an *Ochrobactrum* sp. [7] to increase reaction rate in isomerizing l-rhamnose to l-rhamnulose.

Prompted by this importance and by the rare mentions of biocatalysts of marine origin in specialized reviews, it seems of interest to deepen the knowledge of isomerases from this habitat dedicating a specialized in-depth analysis to them. Indeed, during this literature search, it became evident that curiosity-driven scientific interest has been long-standing as in the case of phosphoglucose isomerases in marine mollusks [8]. This review is dedicated mainly to examples of the applications of marine isomerases, discussing general scientific interest from a historical perspective and in tabulated forms.

## 2. Literature Search

The search for articles in this review has been preliminarily conducted using the words “isomerase” and “marine” in titles, abstracts, and keywords without interval time limit in the Science Direct database with access to 3800 scientific journals in major scientific disciplines. However, the limited range of articles retrieved (31 hits) prompted us to use the more generic PubMed database with the same words thus retrieving 172 hits. Besides, trivial names of these enzymes (as epimerase, racemase, *cis*-*trans* isomerases, cycloisomerase, and tautomerase) were also coupled with “marine” in an accessory search using Web of Science database (Table 1). Science alert mailing lists were used, up to the manuscript submission to update the analysis with the most recent results. In this review, an analysis of reports about enzymatic activities already oriented to applications is firstly compiled in the central Section 3. In Section 4, Section 5 and Section 6, tabulated lists of the remaining articles according to types of molecules on which these isomerases can catalyze their reactions are discussed. Isomerases acting on carbohydrate molecules (Table 2) [9,10,11,12,13,14,15,16,17,18,19,20,21,22,23,24,25,26,27,28,29,30,31,32,33,34,35,36,37,38] are listed in the first of three tables following with lipids (Table 3) [39,40,41,42,43,44,45,46] and amino acids and peptides (Table 4) [47,48,49,50,51,52,53,54,55,56,57,58,59,60,61,62,63,64,65,66,67,68,69,70,71,72,73,74]. Each chronologically ordered line reports the name of the enzyme, as indicated in the reference, the reaction catalyzed, and a short description of the intent and scientific field of the work.

## 3. Application-Oriented Biocatalysts

A few applicative examples of works using marine isomerase are collected in this paragraph.

A gene encoding for d-xylose isomerase from a marine bacterium, *Vibrio* sp. strain XY-214, has been expressed in *E. coli,* and the production of d-xylulose from β-1,3-xylan was carried out. This paper concerns the growing attention to green technologies for the conversion of biomass and saccharification of carbohydrate polymers, in particular, the polysaccharide β-1,3-xylan of the invasive green alga *Caulerpa taxifolia*. Marine *Vibrio* sp. strain can grow on β-1,3-xylan as a sole carbon source; the rationale for the work is based on the synergistic action of two types of enzymes enabling the complete degradation of β-1,3-xylan to d-xylose, i.e., 1,3-β- d-xylan xylanohydrolase and a β-1,3-xylosidase. d-xylulose is then formed by the marine d-xylose isomerase. d-xylulose can be used for ethanol fermentation thus allowing the use of the algal polymer β-1,3-xylan of *C. taxifolia* as a base for ethanol production. The article is a preliminary study for a possible real application of the total saccharification of the polymer. The work has been conducted by purified enzymes, and 2.62 g/L reducing sugars were released by the action of the two β-1,3-xylan degrading activities, the subsequent isomerization of d-xylose thus producing d-xylulose. Time-course experiments analyzing reaction mixtures by HPLC were reported, and different reaction conditions were analyzed also in presence of sodium tetraborate for possible complexes with xylulose-borate shifting the equilibrium [75].

*Yarrowia lipolytica* is a marine microorganism of industrial interest for the physiological ability to utilize different substrates for growth (polyalcohols, organic acids, and long-chain hydrocarbons). In a recent short communication [76], the isomaltulose production using an engineered *Yarrowia lipolytica* strain is reported. Sucrose isomerase catalyzes the enzymatic rearrangement of the α-1,2 linkage between glucose and fructose to an α-1,6 linkage (producing isomaltulose) or α-1,4 linkage (producing trehalulose). Marine origin examples of sucrose isomerase and its use for biological isomaltulose production were not known up to a review of 2014 [77]. In fact, the sucrose isomerase overexpressed from *Pantoea dispersa* but the high and efficient process of isomaltulose production was based on enzyme production and enzymatic catalysis during fermentation, thus reducing costs and simplifying the bioprocess. The maximum isomaltulose production was 572.1 g/L, with a yield of 0.96 g/g of sucrose.

**Figure 1:**
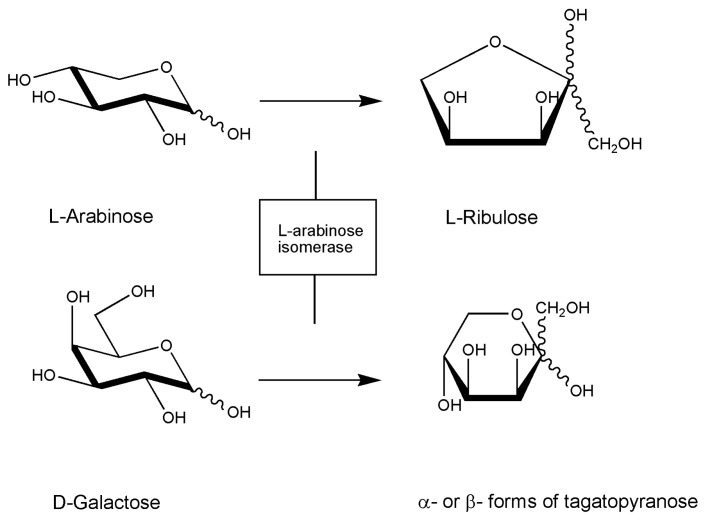
Reaction scheme of l-arabinose isomerase. The enzyme can also convert d-galactose to d-tagatose with lower efficiency. The enzyme is also present in the marine *Geobacillus stearothermophilus* (see Table 2).

A very recent report demonstrates the use of l-arabinose isomerase for production of D-tagatose, a rare sugar of importance in the food industry that has been approved as GRAS drug by the US Food and Drug Administration and used as a substitute for sucrose in low-calorie sweeteners. As in the above case, although the enzyme is from *Lactobacillus sakei* 23K and converts d-galactose from agar into d-tagatose, it is worth to mention this research effort here to show the interest for the profitable application of red algae carbohydrate polymers as a substrate for d-tagatose production (Figure 1) [78].

Ribose-5-phosphate isomerase is another enzyme of interest in the field of rare sugars that are used as sweeteners and for production of interesting building blocks for fine chemistry as reported in the study of ribose-5-phosphate isomerase from an *Ochrobactrum* sp. [7] to increase reaction rate in isomerizing l-rhamnose to l-rhamnulose already mentioned. Although the microorganism was isolated from soil samples, marine examples are also known and could be potential alternatives. Substrate specificity and reaction properties were explored, the results encouraged for the application of ribose-5-phosphate isomerase as a biocatalyst in preparation of rare sugars.

**Figure 2:**
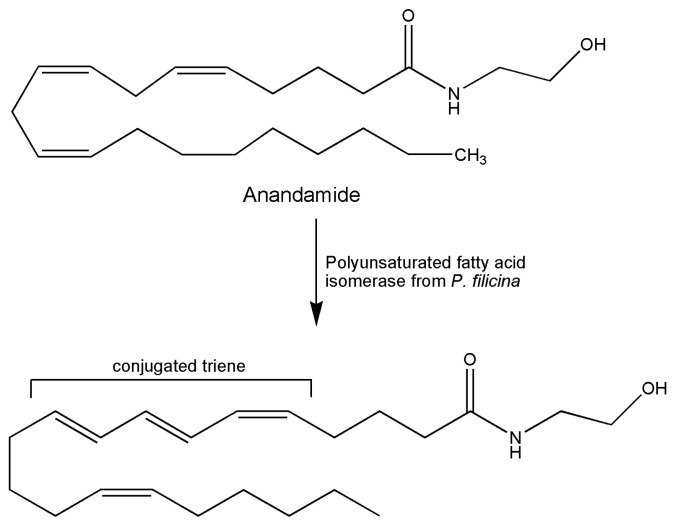
Reaction scheme of formation of conjugated triene anandamide catalyzed by *P. filicina* enzyme.

A report already present in literature in 1994 was the first describing a novel isomerase for the biosynthesis of conjugated triene-containing fatty acids in the red alga *Ptilota filicina* [79]. At the time, in fact, many hypotheses and studies on the biosynthetic pathway of conjugated polyenes in marine organisms were already present in literature, coming from studies centered on natural products of marine origin. The main interest is focused on pharmacological studies indicating a role for these bioactive molecules in the treatment of tumors, against weight gain, and as enhancers of the immune system. In particular, the biosynthesis of a conjugated triene (4,5*Z*,7*E*,9*E*,14*Z*,17*Z*)-eicosapentaenoic acid from eicosapentaenoic acid was indicated. The product is present among natural products of the red alga *Ptilota filicina*. The enzyme was isolated from alga tissues and assayed with arachidonic acid forming a triene structure, evidenced by UV absorption. The product of arachidonate incubation was also identified as the corresponding conjugated triene metabolite. A substrate specificity investigation revealed that the eicosapentaenoic structure was the best substrate for the enzyme. Incorporation of deuterium at C11 position of arachidonate was demonstrated by ^1^H NMR spectroscopy and mass spectrometry for the reaction conducted in the deuterated buffer. Intramolecular and intermolecular hydrogen transfers using stereospecifically deuterated substrates and oxygen sensibility of reaction were also studied. These authors were able to show that unlike the well-characterized aerobic reaction of lipoxygenases, molecular oxygen was not required by their isomerase with no net desaturation occurring during the reaction, thus providing useful insights for the use of these biocatalysts as tools for the synthesis of novel compounds. Later in time, the *P. filicina* enzyme was purified to electrophoretic homogeneity, and the cloning and functional expression including the study of other important characteristics such as molecular weight, subunit structure, and glycosylation were reported [3]. The ability of this enzyme in the isomerization of methylene interrupted olefins led the authors to try the reaction with anandamide, the well-known N-ethanolamide of arachidonic acid, the first endogenous ligand of cannabinoid receptor. The conjugated triene anandamide product was shown to possess high-affinity binding for the receptor [80] (Figure 2). A 33% yield was obtained in preparative experiments for the full spectroscopic chemical characterization of the reaction product. As the same authors speculated, they were able to show the use of these marine enzymes in the synthetic production of novel compounds for pharmacological probes. The same group already studied, in 1991, the oxylipin metabolism. In this work, the conversion of arachidonic acid into the vicinal diol fatty acid 12*R*,13*S*-dihydroxy-5*Z*,8*Z*,10*E*,14*Z*-eicosatetraenoic acid by an acetone powder of the marine red alga, *Gracilariopsis lemaneiformis,* occurred via intermediate formation of hydroperoxide 12*S*-hydroperoxy-5*Z*,8*Z*,10*E*,14*Z*-eicosatetraenoic acid, postulating the existence of a hydroperoxide isomerase in this red alga. The broad substrate specificity and the high stereospecificity of the product formed in the step of oxygen insertion were of interest in the application in biocatalysis [81].

By studying how cultured fish cells derived from turbot (*Scophthalmus maximus*), gilthead seabream (*Sparus aurata*), and Atlantic salmon (*Salmo salar*) metabolize all-cis octadecapentaenoic acid, some authors discovered the action of an isomerase acting on all-*cis* 18:5*n*-3 acids producing 2-*trans* 18:5*n*-3 acids thought to be common intermediates in the β-oxidation of these acids by marine animals [82]. Similar isomerases acting on double bonds of different compounds have been hypothesized in a study on biodegradation of alkenones and related compounds of the marine microalgae *Emiliania huxleyi* by microbial mats collected in large ponds. Among products, authors found *cis*/*trans* or *trans*/*cis* alkene and alkenone isomers and suggested that the formation of these isomeric compounds is likely due to extracellular bacterial *cis*/*trans* isomerases [83].

**Figure 3:**
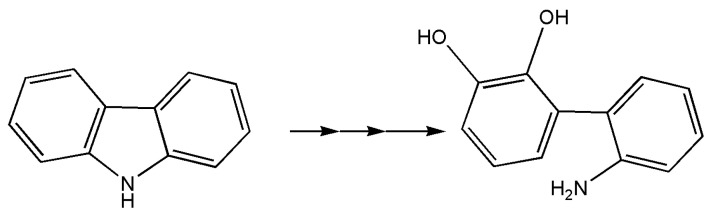
Carbazole degradation to 2′-aminobiphenyl-2,3-diol. Remediation study is present for this compound by the marine bacterium *Neptuniibacter* sp. A tautomerase could be involved in the lower degradation pathway as in total cleavage pathway for the degradation of phenols, modified phenols, and catechols.

In a study centered on searching for marine microorganisms capable of carbazole remediation schematically represented in Figure 3, the marine bacterium *Neptuniibacter* sp. strain CAR-SF has been found utilizing carbazole as its sole carbon and nitrogen sources [84]. Among the enzymes involved in the degradation pathway, 4-oxalocrotonate tautomerase (and others) is indicated, and *Escherichia coli* cells transformed in this work required ferredoxin and ferredoxin reductase for necessary initial dioxygenation of carbazole. The authors indicated that this was the first report of genes involved in carbazole degradation isolated from a marine bacterium, however, only the conversion product of carbazole through dioxygenation by dioxygenase was found (2′-aminobiphenyl-2,3-diol).

**Figure 4:**
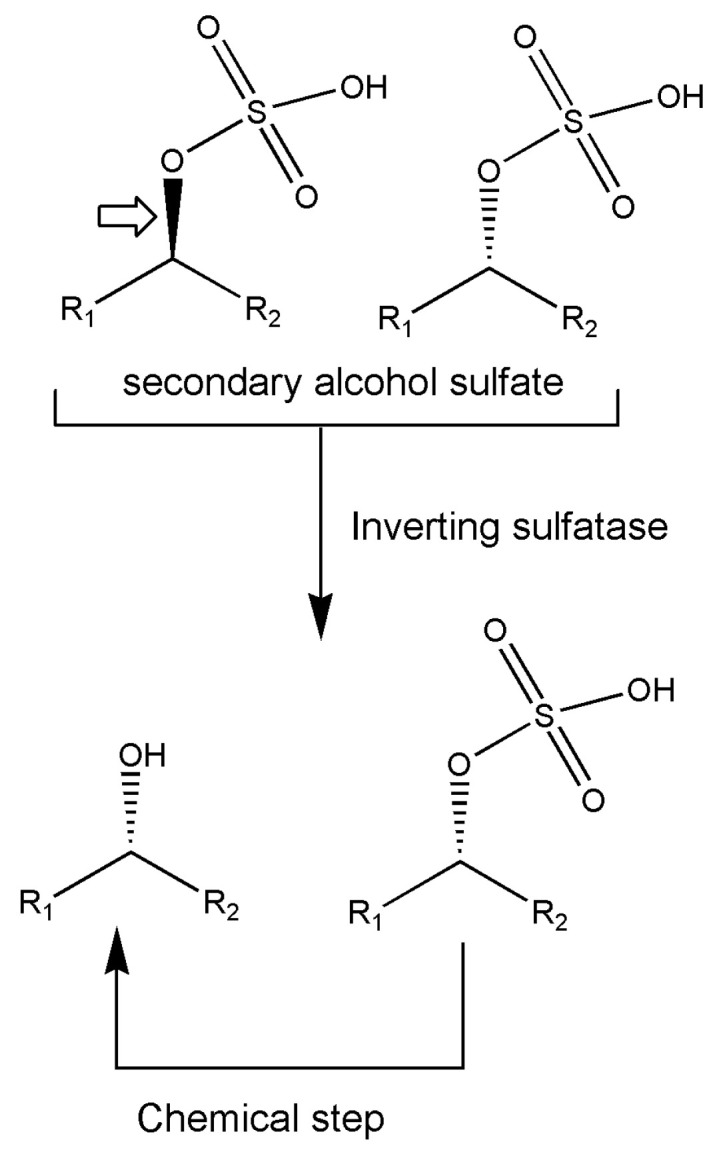
The reaction scheme for the enantioconvergent chemoenzymatic hydrolysis of sulfate esters by inverting marine *Rhodococcus* sulfatase. Big arrow indicated the preferred substrate.

Various enzymes have been analyzed in an interesting report [85] on strategies for the deracemization of a racemate into a single stereoisomeric product; these include mandelate racemase, lactate racemase, or alkyl sulfatases from the actinomycete *Rhodococcus ruber* DSM 44541, the marine planctomycete *Rhodopirellula baltica* DSM 10,527, and others, known to possess a rich inorganic sulfur metabolism. Although not belonging to isomerases, it is worth mentioning that alkyl sulfatases display not only enantioselectivity but also stereoselectivity for retention or inversion of the configuration of the formed product during sulfate hydrolysis and these authors report about a scheme devised to produce single stereoisomer from racemate. The (±)-sec-alkyl sulfate ester is subjected to inverting alkyl sulfatase producing a mixture of hydrolyzed ester with the same configuration of the remaining unreacted ester. The latter is then hydrolyzed in a chemical step with retention of configuration producing the alcohol in this enantioconvergent process (Figure 4). The importance of marine-originating biocatalysts is clearly assessed by this example.

**Figure 5:**
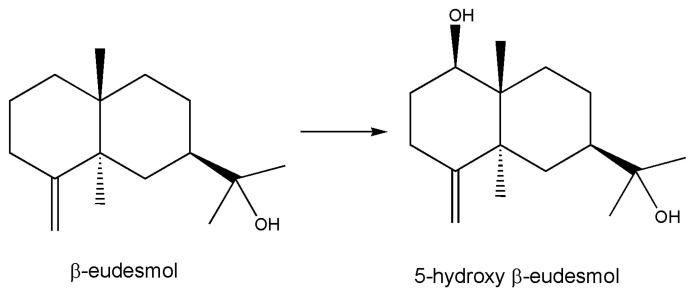
β-eudesmol hydroxylation. Regio- and stereo-specific actions of cytochrome P450 [86] were studied in depth with using NMR spectroscopy; 80% yield in preparative experiments was obtained.

Cytochromes P450 are important biocatalysts performing hydroxylation reactions in regio- and stereospecific manners operating on inactive carbon atoms; they are useful for the bioassisted synthesis of organic molecules. In an interesting paper [86], authors constructed a fusion protein of a peptidyl-prolyl *cis*-*trans* isomerase isolated from the hyperthermophilic archaeon *Thermococcus* sp. with the cytochrome P450 BM3 derived from *Bacillus megaterium* and evaluate its stability in *E. coli* cells in a series of bioconversion experiments with various substituted naphthalenes. It is known that peptidyl-prolyl *cis*-*trans* isomerases catalyze the *cis*-*trans* isomerization of the proline imide bond in polypeptides, which may affect the folding rate of proteins. This fusion protein exists as the predominant soluble protein and more stable than the unfused P450. Various substituted naphthalenes were converted to their monohydroxylated derivatives, and the reaction was also tested on a sesquiterpene (Figure 5) that has physiological functions such as β-eudesmol that was found to be hydroxylated in a regio- and stereo-specific manner.

## 4. Marine Isomerases Acting on Sugar Molecules

**Figure 6:**
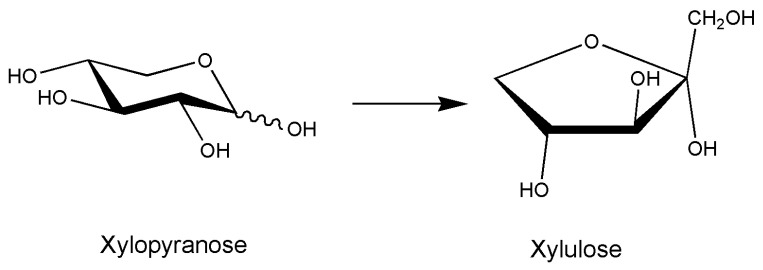
Xylose isomerase reaction; the enzyme catalyzes the interconversion of aldose and ketose sugars with broad substrate specificity; in the case of glucose, fructose is formed, and xylose isomerases are used extensively in the high-fructose corn syrup industry. The hemicellulose fraction of cellulosic biomass can be converted to xylose by xylanases, the need of xylose isomerase is based on the possibility of fermenting d-xylulose by *Saccharomyces cerevisiae* completing biomass utilization, being xylose not fermented.

In Table 2, the articles [9,10,11,12,13,14,15,16,17,18,19,20,21,22,23,24,25,26,27,28,29,30,31,32,33,34,35,36,37,38] found on isomerases acting on carbohydrate pathways are briefly listed. As cited in the first entry about *Pelvetia canaliculata* in 1973 [9], the scientific interest was early generally present in literature since 1956 in investigations conducted on different organisms. For an algal polymannuronic-5-epimerase, converting polymannuronic acid to a mixed polymer containing guluronic acid, the preparation of ammonium sulfate precipitation was reported [9]. Scientific interests for the articles listed in Table 2 and experimental methodologies used reflected the successes in biochemistry, molecular biology, and genetics achieved during the last century. All these listed articles did not contain applicative results that are detailed above in the paragraph on application-oriented biocatalysts. Very often, a basic study of the marine biocatalyst is reported as in the case [28] of xylose isomerase from *Fulvimarina pelagi*. It was identified by sequence analysis of the *F. pelagi* genome, i.e., PCR amplified, cloned, and expressed in *E. coli*, while the aim of the work is framed into the field of biofuel production (Figure 6).

In some cases, the interest of the article is focused on biomedical field as for the two reports [32,33] for the study of allergen function of triosephosphate isomerases from *Octopus fangsiao* and freshwater crayfish *Procambarus clarkii*. The consumption of seafood products, in fact, can be related to the high frequency of food-induced immune responses, and these studies are important to develop therapeutic and diagnostic approaches to these issues. In the case of freshwater crayfish *Procambarus clarkii,* the increased production and consumption can result, in fact, in allergic reactions, including life-threatening anaphylaxis. Another interesting article showing modern scientific interest is the one related to GDP-l-galactose mutase in *Marinactinospora thermotolerans* [34], an article dealing with a rare and interesting molecule, l-Gal*f*, hardly found in the environment; the biosynthetic action and roles and relevant enzymes acting on l-Gal*f* are of interest as this moiety is inserted in an aminonucleoside antibiotic possessing medicinal potential.

## 5. Marine Isomerases Acting on Lipid Molecules

Table 3 contains a few listed articles concerning isomerases acting on lipid molecules. The scientific interest was present as early as the beginning of the 1990s as indicated by the genetic study [39] related to the steroidogenic enzyme involved in the production of 17α-hydroxyprogesterone in the trout *Oncorhynchus mykiss*. The enzyme, produced after expression of cDNA in COS-1 cells, was capable of converting dehydroepiandrosterone to androstenedione. The article, aimed at the finding of necessary probes for identification of steroidogenic enzyme genes in fish species, is in the frame of investigations on the molecular evolution of vertebrate steroidogenic enzymes. As for industrial production of carotenoid pigments such as β-carotene and astaxanthin utilized as food or feed supplements, the interest for the marine bacterium *Agrobacterium aurantiacum* is well documented [40] in the reported review discussing the advances achieved in the field of metabolic engineering for the microbial production of these compounds at the end of 1990s’. In 1999 [42], from the same bacterium, a gene cluster was introduced into *E. coli* to produce astaxanthin at a value 50 times higher than previously achieved. All the aspects about the enzymes of interest from the marine alga *Ptilota filicina* were already discussed in the previous paragraph, and recent interest for *Dunaliella salina* [46] in this field is also present; the latter study provides an insight for induction of β-carotene production in optimized cultivation systems. The case of *Schizochytrium*, the marine fungus producing significant amounts of docosahexaenoic acid (DHA) is of very interest due to positive effects on atherosclerosis, hypertriglyceridemia, hypertension, and cancers of the compound. The paper listed reports of the mechanisms of DHA biosynthesis in *Schizochytrium* constructing and analyzing cDNA library with the possible interesting prospect for new tools to engineer the production of PUFAs [43]. The sterol composition and the related biosynthetic genes were also studied in *Chromera velia* [44], a marine alveolate although the work is framed in the field of basic studies about sterol composition for deriving chemotaxonomic relationships.

## 6. Marine Isomerases Acting on Amino Acids and Peptides

In Table 4, many articles reported concern alanine racemase found in marine organisms. This enzyme, previously discovered in 1951 in other sources was found [47] in the bivalve *Corbicula japonica* in 1985. The authors partially purified the protein evaluating biochemical properties in relation to those of bacterial origin and linked enzyme role to the osmoregulation in these marine organisms. Many other works related to this enzyme are present [2,48,51,52,53] always concerning the osmoregulation action, as in the crayfish *Procambarus clarkii* and in the hepatopancreas of black-tiger prawn, *Penaeus monodon,* up to recent interest for salinipeptins, a group of natural peptides in halotolerant *Streptomyces* isolated from the Great Salt Lake. Salinipeptins, natural products containing D-amino acids, are subjected to extensive enzymatic post-translational modifications during biogenesis [72]. They are substrates of potentially new epimerases of interest during these bioprocesses. A study related to genomic analysis of serine racemase is also found [61]; d-serine, besides frequently found in the bacterial cell walls, lipopeptides and siderophores, also exists as a free molecule in the marine environment with *Roseobacter litoralis* being a special producer. The cases related to disulfide isomerase are also numerous in this Table. They are mostly related to the studies on conotoxins or conopeptides, disulfide-rich peptides found in cone snails that found application in research and possible therapy. The studies mainly based on genetics focus the attention on post-translational reactions catalyzed by these enzymes for diversification of conopeptides structures and folding. Other interests are related to mechanisms of the immune response. Another topic of great interest among these isomerases is related to the cyclophilins (peptidyl-prolyl *cis*-*trans* isomerase, PPIase activity) that catalyze the isomerization of peptide bonds from *trans* to *cis* at proline residues and facilitate protein folding. Their expression is usually enhanced in response to inflammation or malignancy and are involved in functions related to cell metabolism and energy homeostasis and are of therapeutic importance for these and other actions (antifungal, antiviral, and antioxidant activities); they are also of economic importance in oysters cultivation for their involvement in the oyster immune response against infections of *Crassostrea ariakensis* by pathogen rickettsia-like organisms [66].

## 7. Other Enzymes

There are other reports on different enzymatic activities belonging to isomerases that do not fit well into the sections above and are mentioned in this paragraph. The first is an interesting review on chitin metabolism in the marine environment [87]. Authors hypothesized the presence of a mutase in the chitin catabolic cascade, in a more complex system with respect to the usually simplistic accepted hydrolytic pathway based on a chitinase producing the disaccharide N,N′-diacetylchitobiose, and on a beta-N-acetylglucosaminidase producing the final product GlcNAc. The mutase, as described in this review, could represent the activity that converts GlcNAc-1-P, generated from small chitin oligosaccharides and chitobiose for entering the cell membrane, into the 6-P.

Various other articles are present dealing with dopachrome tautomerases involved in the final step of the enzymically regulated melanin biogenesis for the conversion of dopachrome into dihydroxyindoles. In marine organisms, especially in bivalves, the enzymes involved in the biogenesis of melanin are recognized as the general class of phenoloxidases while less is known about the existence and functional role of dopachrome tautomerase genes [88] in mollusk or other organisms [89,90]. However, this field of investigation is quite active due to the role of D-dopachrome tautomerase as cytokine, member of the macrophage migration inhibitory factor protein superfamily. They are associated to important physiological processes such as cell recruitment and migration, tumorigenesis and cancer progress, and inflammatory and autoimmune diseases. Many of these studies on immune system of marine fish may contribute to develop better disease management strategies for fish aquaculture as for Japanese sea bass (*Lateolabrax japonicus*) [91] or for the clam *Ruditapes philippinarum* [92].

## 8. Conclusions

The study of biocatalysts on a global scale from marine environment is just starting and possesses huge potential for the development of applications with industrial benefits due to marine biological diversity and to the specificity of biological marine metabolisms. This knowledge constitutes the core of marine biotechnology and only a deep understanding of the complexity of this ecosystem will enable human beings to protect the oceans and organisms populating them and pave the way for the sustainable exploitation of marine resources. Among the many fields covered that are highly relevant to societal challenges the biorefinery value-chain, food industries and fine chemicals are included among others. However, many challenges remain, generally speaking a deep comprehension of the “marine biotechnology landscape” and a multidisciplinary approach, in education and training [93].

In two comprehensive reports on examples of the application of marine-originating biocatalysts in 2010 and 2016 abovementioned [1,4], marine isomerases were hardly discussed although other classes of enzymes cited are used in food and pharmaceutical applications. After the analysis of literature articles, a first undoubted conclusion of this in-depth review is that curiosity-driven scientific interest for these enzymes seems to be present for a long time. Most of the literature found, tabulated according to the type of molecules on which these enzymes act, indicated a general scientific interest in historical perspective in different fields. As more recent examples, the biomedical field for allergen function of triosephosphate isomerases for seafood consumption, or efforts for the elucidation of the biosynthetic action of GDP-L-galactose mutase acting on interesting and rare L-Gal*f*, must be mentioned. As for isomerases acting on lipid molecules, both basic interest for investigations on the molecular evolution of vertebrate steroidogenic enzymes or more oriented studies for carotenoid pigments production, are present. Similar situation for isomerases acting on protein molecules was noted, e.g., of alanine racemases, in the studies related to the role of the osmoregulation in marine organisms and for new epimerases catalyzing interesting bioprocesses during post-translational modifications of natural peptides known as salinipeptins.

On the other hand, application-oriented examples of marine isomerases already applied in biocatalysis are a few confirming the general result reported in previously published reviews [1,4]. However, major fields in which these few papers are framed are depicted in a better manner in this review. Works are placed at the cutting edge of biotechnological development such as the conversion of biomasses and saccharification of carbohydrate polymers (d-xylose isomerase), in biomedicine and nutraceuticals (isomaltulose production, l-arabinose isomerase for production of d-tagatose and ribose-5-phosphate isomerase), and in bioremediation field (cytochromes P450, carbazole remediation, etc.). Therefore, despite the scarcity of direct applicative examples found, novel stability features and chemical/stereochemical properties found in general examples of marine biocatalysts will be present in the numerous studied isomerases as well. These enzymes in fact can offer properties related to the habitat, which are greatly appreciated under a general biotechnological perspective. As last conclusion, it can be said that these properties will surely act as a promoter for future studies of these marine-originating isomerases in applied biocatalysis.

## Figures and Tables

**Table 1:** Searches in literature.

Databases	Search Statement	Hits
Science Direct	Isomerase * and marine in titles, abstracts and keywords	31
PubMed	Isomerase * and marine	172
WoS ^1^	Marine epimerase * in All fields	53
WoS	Marine racemase * in All fields	44
WoS	Marine cis–trans isomerase * in All fields	56
WoS	Marine cycloisomerase * in All fields	3
WoS	Marine tautomerase * in All fields	26
WoS	Marine mutase * in All fields	45

^1^ Web of Science, last date accessed June 2020 with updates up to submission; * indicated the plural.

**Table 2:** Marine isomerases acting on sugar molecules.

Reference/Year	Organism	Enzyme	Reaction	Note
[9] 1973	Alga *Pelvetia canaliculata*	Polymannuronic-5-epimerase	Conversion of polymannuronic acid to a mixed polymer containing guluronic acid	Preparation of ammonium sulfate fraction of the enzyme
[10] 1977	Marine species of *Alcaligenes*, *Pseudomonas marina*, and *Alteromonas communis*	P-hexose isomerase	Glycolytic pathway	Entner–Doudoroff pathway
[11] 1984	Marine snails	Phosphoglucose isomerase	Glycolytic enzyme	Tolerance to detergents as monitoring tool
[12] 1985	Bivalve mollusks: *Mytilus edulis* and *Isognomon alatus*	Glucose phosphate isomerase	Glycolytic enzyme	Biochemical-based study of adaptation of enzyme to temperature
[13] 1995	Psychrophilic marine eubacterium *Vibrio* sp. strain ANT-300	Triosephosphate isomerase	Interconversion dihydroxyacetone phosphate and d-glyceraldehyde-3-phosphate	Thermolability study
[14] 1995	Marine red alga *Gracilaria verrucosa*	Triosephosphate isomerase	Interconversion dihydroxyacetone phosphate and d-glyceraldehyde-3-phosphate	Genetic study
[15] 2001	Polychaeta *Polydora brevipalpa*	Glucose-6-phosphate isomerase	Glycolytic enzyme	Study of isozyme pattern
[16] 2001	Marine ammonia-oxidizing bacteria *Nitrosomonas*	Triosephosphate isomerases	Interconversion dihydroxyacetone phosphate and d-glyceraldehyde-3-phosphate	Purification and characterization
[17] 2003	Macroalga *Solieria chordalis*	UDP-glucose-4-epimerase	Catalyzing both the synthesis of UDP-Gal and UDP-Glc	Characterization of the enzyme
[18] 2008	Blue mussel *Mytilus edulis*	Mannose-6-phosphate isomerase	Glycolytic enzyme	Genetic study
[19] 2010	Marine *Geobacillus stearothermophilus*	l-Arabinose Isomerase	Converting d-galactose to d-tagatose	Clone and sequence araA gene
[20] 2012	Marine copepod *Tigriopus californicus*	Phosphoglucose isomerase	Glycolytic enzyme	Genetic variability study
[21] 2012	*Thermotoga maritima*	Tagaturonate-fructuronate epimerase UxaE	Epimerization of tagaturonate to fructuronate	Study of metabolism of galacturonate and glucuronate from pectin and xylan
[22] 2012	*Pyrococcus horikoshii*	UDP-glucose 4-epimerase	Catalyzing both the synthesis of UDP-Gal and UDP-Glc	Characterization study of the enzyme that could be coupled with trehalose synthase
[23] 2013	Marine bacterium *Bermanella marisrubri* sp. RED65	d-glucuronyl C5-epimerase	Epimerization of d-glucuronic acid to its C5-epimer l-iduronic acid	Recombinant protein expressed in *Escherichia coli* showed epimerization activity
[24] 2014	Brown algae	Alginate-C5-mannuronan-epimerase	Catalyze the conversion of mannuronate to guluronate and determine the M/G ratio of alginate	Genetic study: predicted 94 algG genes open reading frame (ORF) sequences of brown algae
[25] 2015	Marine bacterium *Vibrio* sp.	3,6-Anhydro-l-galactonate cycloisomerase	Converts 3,6-anhydro-l-galactonate into 2-keto-3-deoxygalactonate	Identification of intermediate products of 3,6-anhydro-l-galactose
[26] 2015	*Pyrococcus horikoshii*	Phosphomannose isomerase	Mannosylglycerate biosynthetic pathway	Recombinant protein expressed in *E. coli* with double activity (Man-1-P GTase activity)
[27] 2016	Marine Pacific whiteleg shrimp *Litopenaeus vannamei*	Triosephosphate isomerase	Interconversion dihydroxyacetone phosphate and d-glyceraldehyde-3-phosphate	Structural and mechanistic study and insights into glycolysis regulation in crustaceans
[28] 2016	Marine bacterium *Fulvimarina pelagi*	Xylose isomerase	Interconversion of d-xylose and d-xylulose	Cloning, expression, and characterization for use in biofuels’ production
[29] 2016	Brown alga *Ectocarpus*	Mannuronan C5-epimerase	Control the distribution pattern of (1-4) linked β-d-mannuronic acid (M) and alpha-l-guluronic acid (G) residues in alginates	Transcript expression
[30] 2016	Alga *Saccharina japonica*	Mannuronan C5-epimerase	Control of the distribution pattern of (1-4) linked β-d-mannuronic acid (M) and alpha-l-guluronic acid (G) residues in alginates	Functional recombinant expression of protein in insect-cell system revealing alternate epimerization of beta-d-mannuronic acid to alpha-l-guluronic acid
[31] 2017	*Vibrio* sp. strain EJY3	3,6-Anhydro-l-galactonate cycloisomerase	Converts 3,6-anhydro-l-galactonate into 2-keto-3-deoxygalactonate	Crystallization and X-ray analysis of recombinant protein
[32] 2017	*Octopus fangsiao*	Triosephosphate isomerase	Interconversion dihydroxyacetone phosphate and d-glyceraldehyde-3-phosphate	Study of allergen function
[33] 2017	Freshwater crayfish *Procambarus clarkii*	Triosephosphate isomerase	Interconversion dihydroxyacetone phosphate and d-glyceraldehyde-3-phosphate	Study of allergen function
[34] 2017	*Marinactinospora thermotolerans*	GDP-l-galactose mutase	Conversion of pyranose form to furanose structure	Study of the sugar biosynthetic pathway
[35] 2018	Marine fungus-like thraustochytrids	Xylose isomerase	Interconversion of d-xylose and d-xylulose	Identification and characterization of xylose metabolism
[36] 2019	*Scylla paramamosain*	Triosephosphate isomerase	Interconversion dihydroxyacetone phosphate and d-glyceraldehyde-3-phosphate	Crystal structure
[37] 2019	*Gracilariopsis lemaneiformis*	Mannose-6-phosphate isomerase, GDP-mannose-3,5-epimerase	Pathways of floridean starch	Transcriptomic study for the study of the mechanism of substrate competition of synthesis pathways of floridean starch
[38] 2020	Marine *Streptomyces lividans* RSU26	Glucose isomerase	Fructose to glucose conversion	Characterization study and optimization of enzyme production

**Table 3:** Marine isomerases acting on lipid molecules.

Reference/Year	Organism	Enzyme	Reaction	Note
[39] 1993	Rainbow trout *Oncorhynchus mykiss*	3β-hydroxysteroid dehydrogenase/Δ(5-4)-isomerase	Steroidogenic enzymes involved in the production of 17α-hydroxyprogesterone	Genetic study
[40] 1997	Marine bacterium *Agrobacterium* *aurantiacum*	Carotenoid gene cluster	β-carotene biosynthesis	Metabolic engineering study
[41] 1997	Alga *Ptilota filicina*	Polyenoic fatty acid isomerase	Assay by conversion of arachidonic acid to a conjugated triene	Biochemical study of binding site characteristics
[42] 1999	Marine bacterium *Agrobacterium aurantiacum*	Isopentenyl diphosphate (IPP) isomerase and gene cluster (crtBIYZW)	Isoprenoid pathway	Study to enhance astaxanthin production by engineering isoprenoid pathway
[3] 2002	Marine alga *Ptilota filicina*	Polyenoic fatty acid isomerase	Assay by conversion of arachidonic acid to a conjugated triene	Study of protein characterization and functional expression
[43] 2008	Marine fungus *Schizochytrium*	Enzymes involved in biosynthesis of fatty acid via polyketide synthases	Confirmation PKS pathway	Genetic study of docohexanoic acid biosynthesis
[44] 2012	Marine alveolate *Chromera velia*	Isopentenyl diphosphate Δ-isomerase	Sterol biosynthesis	Study of sterol composition of *Chromera velia* for chemotaxonomic relationships
[45] 2019	Marine thraustochytrid *Aurantiochytrium*	Isopentenyl pyrophosphate isomerase	Biosynthetic pathways of docosahexaenoic acid (DHA) and ketocarotenoid astaxanthin	Analyses of the genome, transcriptome, key enzymes, and pathway products
[46] 2020	*Dunaliella salina*	15-*cis*-*Z*-carotene isomerase, prolycopene isomerase	β-carotene biosynthesis	Study of β-carotene biosynthesis: seven full length cDNA sequences cloned

**Table 4:** Marine isomerases acting on amino acids and peptides.

Reference/Year	Organism	Enzyme	Reaction	Note
[47] 1985	Bivalve *Corbicula japonica*	Alanine racemase	l to d alanine	Partial purification and characterization
[48] 1992	Eighteen molluscan species	Alanine racemase	l to d alanine	Comparative study and distribution
[49] 1995	*Haloferax volcanii* and *Haloarcula* species	Lactate racemase	l to d lactate	Study of enzymatic diversity among species
[50] 1997	*Halobacterium cutirubrum*	Peptidyl-prolyl *cis*/*trans* isomerase	Isomerization of peptide bonds (*trans-cis*) at Pro residues; facilitates protein folding	Genetic study and expression in *E. coli*
[51] 2000	Crayfish *Procambarus clarkii*	Alanine racemase	l to d alanine	Isolation, kinetic properties, substrate specificity, structural characteristics
[2] 2001	Black-tiger prawn, *Penaeus monodon*	Alanine racemase	l to d alanine	Kinetic properties and substrate specificity
[52] 2005	Microalga *Thalassiosira* sp.	Alanine racemase	l to d alanine	Kinetic properties and substrate specificity
[53] 2006	Marine gastropod *Cellana grata*	Alanine racemase	l to d alanine	First purification study and kinetic assessment in gastropod
[54] 2011	Marine cone snails	Disulfide isomerase	Oxidation, isomerization, and reduction of S–S bonds	Proteomic study showing presence of multitude of isoform of the enzyme
[55] 2012	Channel catfish *Ictalurus punctatus*	Disulfide isomerase	Oxidation, isomerization and reduction of S–S bonds	Genetic study
[56] 2013	Marine alga *Ulva lactuca*	Disulfide isomerase	Oxidation, Isomerization, and reduction of S–S bonds	Study of cloning and expression
[57] 2013	Crab *Eriocheir sinensis*	Peptidyl-prolyl *cis*/*trans* isomerase	Isomerization of peptide bonds (*trans-cis*) at Pro residues; facilitates protein folding	Purification of recombinant protein and assessment of antifungal properties
[58] 2014	Marine bacterium *Vibrio anguillarum*	Peptidyl-prolyl *cis*/*trans* isomerase	Isomerization of peptide bonds (*trans*-*cis*) at Pro residues; facilitates protein folding	Changes in protein expression of *V. anguillarum*, gene expression in *E. coli* and biochemical characterization
[59] 2015	Core snails	Disulfide isomerase	Oxidation, Isomerization, and reduction of S–S bonds	Proteomic study
[60] 2016	Marine *Alphaproteobacteria*	A novel family of peptidyl-prolyl isomerase	Isomerization of peptide bonds (*trans*-*cis*) at Pro residues; facilitates protein folding	Structural and functional characterization
[61] 2016	Marine heterotrophic bacterium *Roseobacter litoralis*	Serine racemase	Racemization and minor dehydration of serine	Genomic analysis
[62] 2016	Thermophilic chlorophycean microalga, *Scenedesmus* sp.	Peptidyl-prolyl *cis*/*trans* isomerase	Isomerization of peptide bonds (*trans*-*cis*) at Pro residues; facilitates protein folding	Cloning and expression of the enzyme in *E. coli* and indication of role in stress-tolerance mechanisms
[63] 2016	Marine thaumarchaeote *Nitrosopumilus maritimus*	Peptidyl-prolyl *cis*/*trans* isomerase	Isomerization of peptide bonds (*trans*-*cis*) at Pro residues; facilitates protein folding	A protein structure study
[64] 2016	Superfamily *Conoidea*	Disulfide isomerase	Oxidation, Isomerization, and reduction of S–S bonds	Study of diversification of enzymatic protein folding correlated with diversity of conotoxins
[65] 2016	Marine snails belonging to *Conus*	Disulfide isomerase	Oxidation, Isomerization, and reduction of S–S bonds	Transcriptomic and in silico analysis and characterization of the group of PDI protein sequences
[66] 2016	Oyster *Crassostrea ariakensis* Gould	Peptidyl-prolyl isomerase (cyclophilins)	Isomerization of peptide bonds (*trans*-*cis*) at Pro residues; facilitates protein folding	Enzymatic tissue distribution and role of the three enzymes identified and involvement in in oyster immune response
[67] 2017	Cone snail species	Disulfide isomerase	Oxidation, Isomerization, and reduction of S–S bonds	Cloned 12 disulfide isomerase genes and study of reaction on conopeptides
[68] 2017	Shrimp, *Litopenaeus vannamei*	Peptidyl-prolyl isomerase (cyclophilins)	Isomerization of peptide bonds (*trans*-*cis*) at Pro residues; facilitates protein folding	Cloning and tissue distribution of the enzyme
[69] 2017	Red alga *Pyropia seriata*	Peptidyl-prolyl isomerase (cyclophilins)	Isomerization of peptide bonds (*trans*-*cis*) at Pro residues; facilitates protein folding	Transcriptomic study
[70] 2018	Cyanobacterial genomes	PoyD, a member of the radical S-adenosylmethionine superfamily	Introducing d-amino acids into a ribosomally synthesized peptide	Heterologous expression in *E. coli*, detection of epimerase activity, and localization of epimerization sites
[71] 2019	Cyanobacteria	AerE, a cupin superfamily enzyme	1,3-allylic isomerization	Study of the biosynthesis of aeruginosins trapeptides possessing antithrombotic activity
[72] 2019	Halotolerant *Streptomyces* sp. strain GSL-6C	Inferring new epimerases	Conversion of l- to d-amino acids	Genome analysis integrating a study on salinipeptins
[73] 2019	Hydrothermal vent mussel *Bathymodiolus azoricus*	Peptidyl-prolyl *cis*/*trans* isomerase	Isomerization of peptide bonds (*trans*-*cis*) at Pro residues; facilitates protein folding	Study of proteome changes upon Cd exposure for bioindicator identification
[74] 2020	Marine red algae *Pyropia yezoensis*	Peptidyl-prolyl isomerase (cyclophilins)	Isomerization of peptide bonds (*trans*-*cis*) at Pro residues; facilitates protein folding	Analysis of the biological activity of recombinant cyclophilin
